# Loss of NEIL3 DNA glycosylase markedly increases replication associated double strand breaks and enhances sensitivity to ATR inhibitor in glioblastoma cells

**DOI:** 10.18632/oncotarget.22896

**Published:** 2017-12-04

**Authors:** Alex W. Klattenhoff, Megha Thakur, Christopher S. Chu, Debolina Ray, Samy L. Habib, Dawit Kidane

**Affiliations:** ^1^ Division of Pharmacology and Toxicology, College of Pharmacy, The University of Texas at Austin, Dell Pediatric Research Institute, Austin, TX, United States; ^2^ South Texas Veterans Health System and Department of Cellular and Structural Biology, The University of Texas Health Science Center, San Antonio, TX, United States

**Keywords:** DNA glycosylase, ATR, replication stress

## Abstract

DNA endonuclease eight-like glycosylase 3 (NEIL3) is one of the DNA glycosylases that removes oxidized DNA base lesions from single-stranded DNA (ssDNA) and non-B DNA structures. Approximately seven percent of human tumors have an altered NEIL3 gene. However, the role of NEIL3 in replication-associated repair and its impact on modulating treatment response is not known. Here, we report that NEIL3 is localized at the DNA double-strand break (DSB) sites during oxidative DNA damage and replication stress. Loss of NEIL3 significantly increased spontaneous replication-associated DSBs and recruitment of replication protein A (RPA). In contrast, we observed a marked decrease in Rad51 on nascent DNA strands at the replication fork, suggesting that HR-dependent repair is compromised in NEIL3-deficient cells. Interestingly, NEIL3-deficient cells were sensitive to ataxia–telangiectasia and Rad3 related protein (ATR) inhibitor alone or in combination with PARP1 inhibitor. This study elucidates the mechanism by which NEIL3 is critical to overcome oxidative and replication-associated genotoxic stress. Our findings may have important clinical implications to utilize ATR and PARP1 inhibitors to enhance cytotoxicity in tumors that carry altered levels of NEIL3.

## INTRODUCTION

Base excision repair (BER) is the main guardian against DNA damage due to cellular metabolism, including lesions resulting from reactive oxygen species, methylation, deamination and hydroxylation [1]. DNA glycosylases remove modified DNA bases from DNA by hydrolyzing the glycosidic, leaving behind an apurinic/apyrimidinic (AP) site [2], which is further processed by AP-endonuclease, DNA polymerases, and DNA ligase activities to restore the original DNA sequence [3]. Five types of DNA glycosylases have been identified in human cells that are responsible for oxidative DNA damage repair: OGG1, NTH1, NEIL1, NEIL2, and NEIL3 [4]. NTH1 [5, 6] and OGG1 specifically remove oxidized purines and pyrimidines from duplex DNA, respectively [7, 8]. DNA endonuclease eight-like glycosylase 1 (NEIL1) acts in concert with the replication fork to remove the lesions before they are encountered by the DNA polymerases [9, 10]. NEIL2 acts during transcription-coupled repair [11, 12].

DNA endonuclease eight-like glycosylase 3 (NEIL3) is one of the DNA glycosylases with an N-terminal glycosylase domain and an uncharacterized C-terminal domain. A recent report has demonstrated that NEIL3 glycosylase activity is critical for embryonic development [13]. Further, NEIL3 is important to maintain stem cell proliferation and differentiation [14]. Human NEIL3 is expressed in the thymus, testes [15], and at high levels in tumor tissues [14, 16, 17]. In contrast, NEIL3 deficiency delayed astrocyte differentiation, inhibited cell cycle progression, and impaired the ability to repair the hydantoin products, spiroiminodihydantoin (Sp) and 5-guanidinohydantoin (Gh) lesions in ssDNA [14]. Further, aberrant function of NEIL3 is associated with increased lymphocyte apoptosis, autoantibodies, and predisposition to autoimmunity [18].

Although there is much redundancy in substrate specificity, cells lacking DNA glycosylase functions show increased levels of DNA base damage, elevated mutation rates, and hypersensitivity to specific DNA damaging agents [19]. NEIL3 prefers substrates that contain single-stranded DNA regions such as looped DNA structures, quadruplexes, and structures representing replication forks [13]. In addition, since NEIL3 has a very weak lyase, and the AP sites remaining after NEIL3 glycosylase activity is likely cleaved by APE1. NEIL3 cell-cycle-dependent expression patterns show induction in the early S phase with peak levels in the G2 phase [16]. Several previous studies suggested that NEIL1/2 plays a role in replication-associated repair or transcription-coupled repair respectively [20, 21]. Replication fork stalls during S-phase are known to instigate subsequent replication fork collapses and induce genomic instability [22–24].

However, the role of NEIL3 in maintaining genomic stability at the replication fork is still unknown. We tested our hypothesis that NEIL3 is required to prevent oxidative and DNA replication-associated DNA damage, and that the loss of NEIL3-related DNA repair function in cancer cells alters the chemotherapy response. Thus, we examined the role of NEIL3 during oxidative and replication stress-associated DNA damage and its impact on DNA damage responses. This study shows that NEIL3 is localized at DSB sites and is associated with the replication fork during oxidative and replication stress. Further, our data shows that the loss of NEIL3 leads to decreased replication fork speed and increased replication-associated DSBs, which exhaust RPA levels in cancer cells and compromised HR dependent repair. Previous studies have shown that cancer cells deficient in DSB repair are sensitive to PARP1 inhibitors [25–27]. Our results show that an inhibitor of PARP1 (Olaparib) sensitizes NEIL3 deficient cancer cells. Moreover, NEIL3 deficient cells are hypersensitive to ATR inhibitor. The reports of the potent sensitizing effect of ATR inhibitor prompted us to assess the effect of the Combinatorial effect of ATR inhibitor and Olaparib on NEIL3 deficient cells. When NEIL3 expression was inhibited by shRNA-NEIL3, synergistic effects were noted between ATR and Olaparib. Simultaneous inhibition of ATR and PARP1 sensitizes NEIL3 deficient cells. As such, the observed sensitization effect of both inhibitors utilized with NEIL3 deficient cancer cell lines may be related to the combined inhibition of both pathways. Our results further suggest that NEIL3 status should routinely be assessed in several cancer types, including glioblastoma, that likely provide a novel therapeutic opportunity to target tumors that carry altered levels of NEIL3.

## RESULTS

### NEIL3 is recruited to sites of DSBs

**Figure 1:**
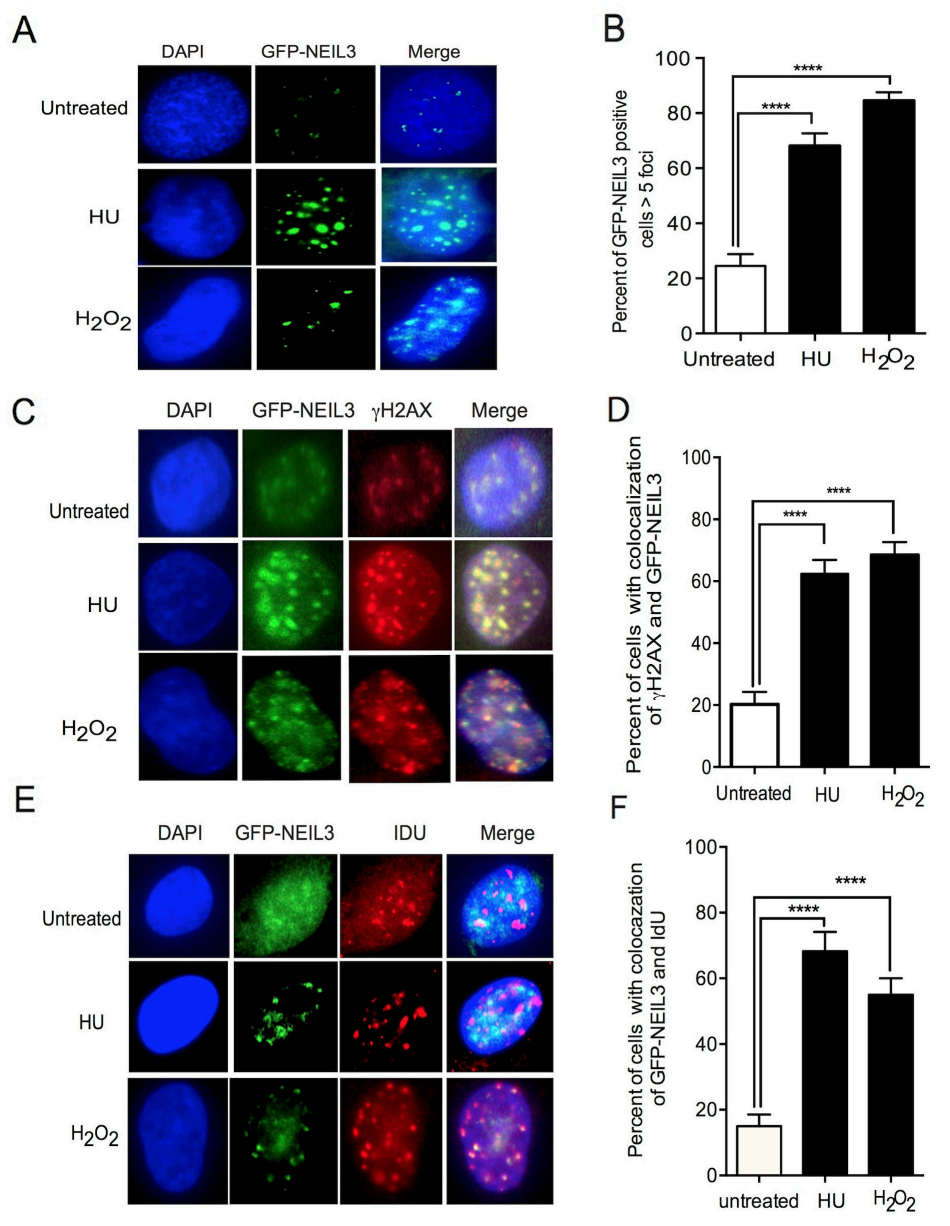
NEIL3 localized at DSB sites and associated with replication foci **(A)** Nuclear localization of GFP-NEIL3 before or after HU/ H_2_O_2_ treatment; **(B)** Estimated percent of cells positive for GFP-NEIL3 before (n= 117) and after HU (2mM) (n=105) / H_2_O_2_ treatment (1000nM) (n=135); **(C)** Co-localization of GFP-NEIL3 and γH2AX before and after HU or H2O2 treatment; **(D)** Quantification of GFP-NEIL3 and γH2AX co-localization using percentage of dual-positive cells between HU treated (n= 117) and untreated groups (n= 106) versus those treated with H_2_O_2_ (n=135); **(E)** Representative images of GFP-NEIL3 and IdU co-localization before and after HU or H_2_O_2_ treatment; **(F)** Quantification of GFP-NEIL3 and IdU co-localization using percentage of dual-positive cells between HU treated (n= 87), H_2_O_2_ treated (n=135) and untreated groups (n= 67).

To determine whether NEIL3 is recruited to the site of DNA damage during oxidative DNA damage and replication stress, we generated cell lines expressing GFP-NEIL3. We induced oxidative and replication-associated DNA damage by treating NEIL3 proficient and deficient LN428 cells with H_2_O_2_ (1000nM) and hydroxyurea (2mMHU), which causes oxidative DNA damage and rapid depletion of dNTPs respectively [28]. Cells were treated with H_2_O_2_ for 1 hour or treated with 2 mM HU for 2 hours and then examined for subcellular localization of GFP-NEIL3. The number of cells that exhibited GFP-NEIL3 nuclear foci were significantly increased in cells treated with H_2_O_2_ or HU versus untreated cells (Figure 1A and 1B; Mean ± SEM, 84±3 or 68 ± 5 versus 24.5±4.2; P=0.0001). Next, we determined the co-localization of GFP-NEIL3 with γH2AX upon treatment of the cells with H_2_O_2_ or HU, as an indicator of the presence of NEIL3 at the DSB sites. Indeed, the number of cells with γH2AX co-localized with GFP-NEIL3 was significantly increased in H_2_O_2_ or HU treated cells versus untreated cells, suggesting that NEIL3 is associated with DSBs (Figure 1C and 1D; 68± 4 or 62 ± 4 versus 20±4; P=0.0001). Next, to determine whether GFP-NEIL3 is associated with active DNA replication, we performed co-immunostaining of the cells with antisera against IdU and γH2AX. The number of GFP-NEIL3 co-localized with IdU were significantly increased in H_2_O_2_ or HU treated cells compared to untreated cells (Figure 1E and 1F; 55±5 or 68± 6 versus 15±4 P=0.0001), suggesting that NEIL3 is associated with DSBs during DNA replication in the S-phase of the cell cycle.

### Oxidative and replication stress-associated DSBs increase in NEIL3 deficient cells

**Figure 2:**
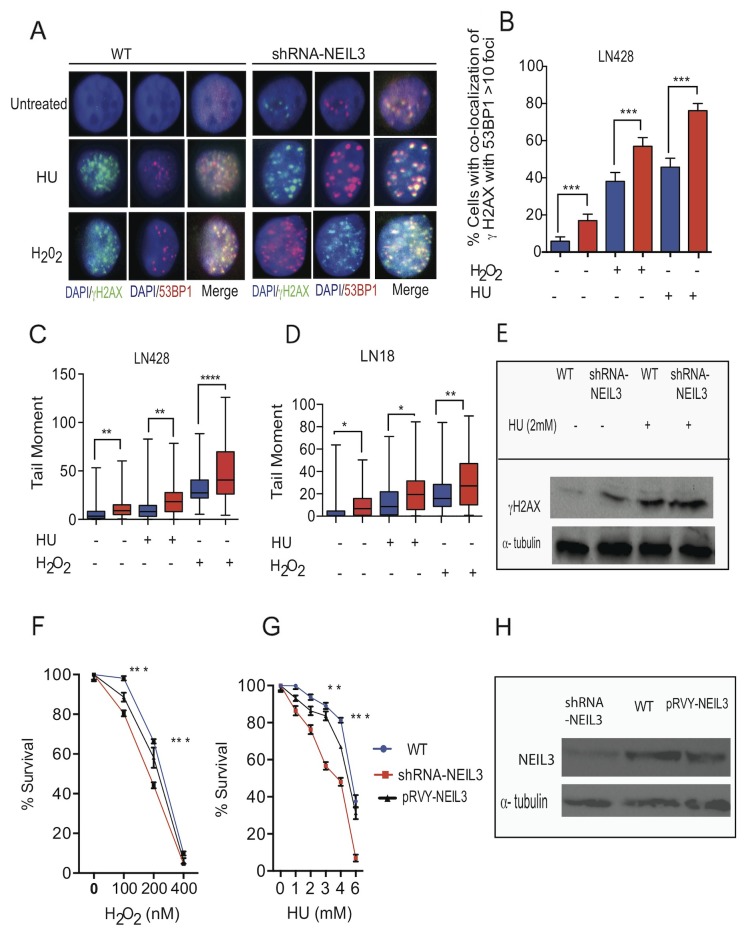
Loss of NEIL3 increases DSBs **(A)** Representative images of DSBs in NEIL3-proficient and NEIL3-deficient cells with or without HU (2mM) or H_2_O_2_ (1000nM); **(B)** Quantified percentage of co-localization of 53BP1 and γH2AX (> 5 foci) before and after HU or H_2_O_2_ treatment in LN428 cells. Note that the number of untreated LN428 proficient (n=100) and deficient (n=100) versus HU and/or H_2_O_2_ treated LN428 NEIL3 proficient (n=100; and 110) and deficient cells (n=110; 150); **(C)** Quantified tail moment of neutral comet assay for LN428 NEIL3 proficient versus deficient cells before (n=92; n=94) and after HU (n=86; 67) or H_2_O_2_ treatment (n=81; 83) included for analysis; **(D)** Quantified tail moment of neutral comet assay to measure DSBs in LN18 NEIL3 proficient and deficient cells untreated (n=59; 48) versus treated with HU (n=41; 63) or H_2_O_2_ (n=70; 73); **(E)** Western blot for γH2AX for LN428 treated with HU (2mM); **(F-G)** Clonogenic cell survival in LN428 NEIL3 proficient and deficient cells with different concentration of H_2_O_2_ treatment for 4 hours (100nM, 200nM, 400nM) (F); with different concentrations of HU treatment (1, 2, 4 and 6 mM) for 2 hours (G); **(H)** Western blot for NEIL3 in LN428 cell lines knockdown but complemented by pRVY-NEIL3 plasmid. All data analysis were performed using GraphPad Prism statistical software.

**Figure 3:**
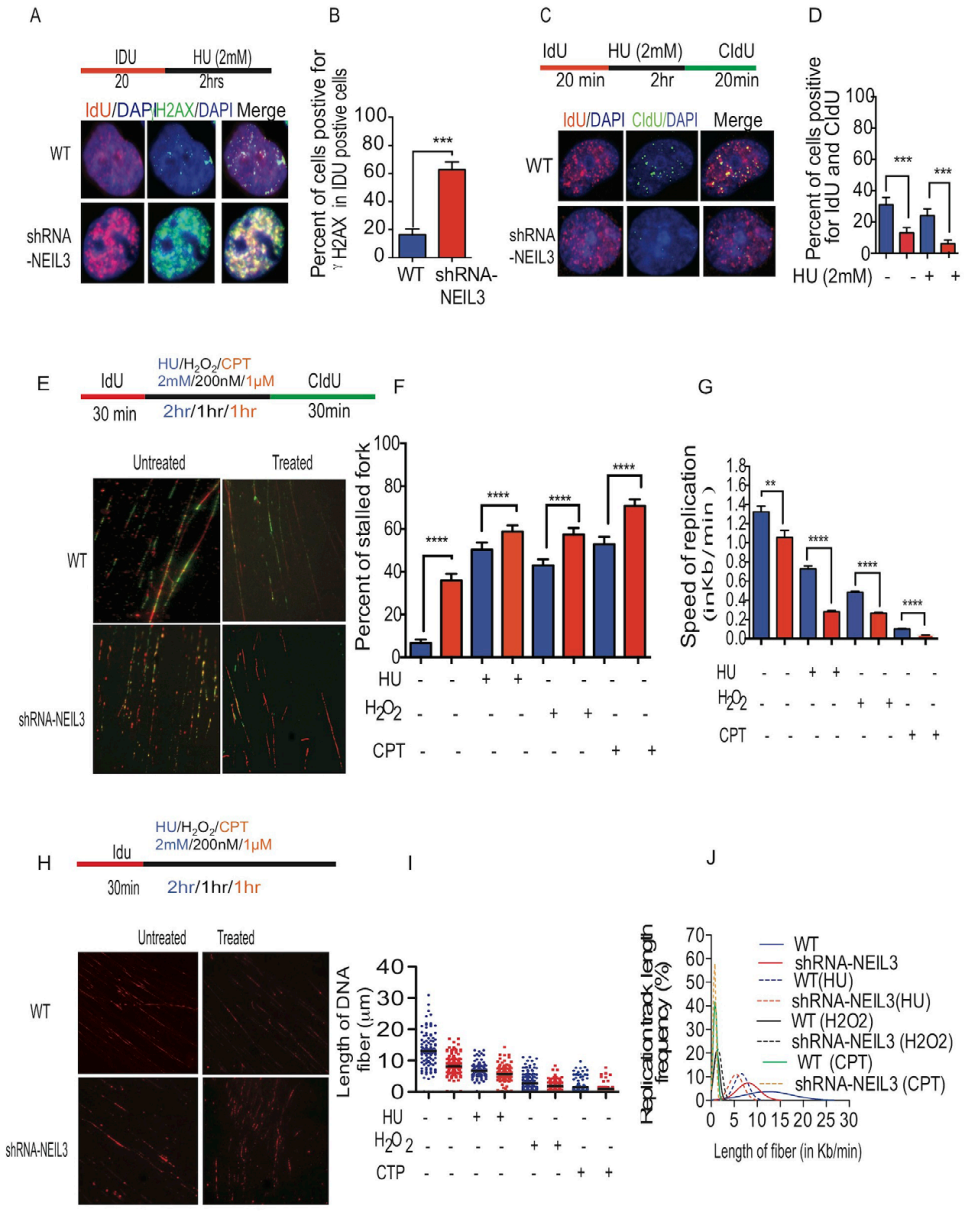
NEIL3 is required for replication fork progression **(A)** Representative images of γH2Ax and IdU colocalization in NEIL3-proficient and NEIL3-deficient cells; **(B)** Estimated percent of γH2Ax positive cells in IdU positive cells in NEIL3 proficient (n=91) and NEIL3-deficient cells (n=75); **(C)** Images of IdU and CIdU co-localization after HU treatment in NEIL3-proficient and NEIL3-deficient cells; **(D)** Estimated number of cells dual-positive for IdU and CIdU. Note that the number of untreated NEIL3 proficient (n=127) and deficient (n=101) versus treated NEIL3 proficient (n= 125) and NEIL3 deficient cells (n=114); **(E)** Representative images of replication fibers from NEIL3 proficient and NEIL3-deficient cells after pulse-labeling with 5 μM IdU for 30 min (red track) and 250 μM CIdU for 30 min (green track); **(F)** Estimated percentage of stalled replication forks in untreated NEIL3-proficient (n=210) and NEIL3-deficient cells (n=226) versus HU treated NEIL3 proficient (n=205) and deficient cells (n=197); H_2_O_2_ treated NEIL3 proficient and deficient (n=282; 263); cisplatin (1μM) treated NEIL3 proficient and deficient cells (n=208; 226); **(G)** Estimated replication fork speed in NEIL3-proficient and NEIL3-deficient cells before (n=169;151) and after HU (n=172; 119), H_2_O_2_ (n=302; 159) or cisplatin treatment (n=348; 159). Note that replication fork speed was calculated by dividing the length of each CIdU track (green) by its incubation time (30 min). **(H)** Schematic representation of DNA fibers from NEIL3 proficient versus NEIL3 deficient cells and images of the visualized nascent DNA strand after treatment. Note that cells were pulsed with IdU for 30 minutes followed by 2mM HU treatment for 2 hours, or H_2_O_2_ (1000nM) for 1 hour; cisplatin (CTP; 1μM) for 1 hour then DNA fiber experiment were done as described previously (upper panel); **(I)** Estimated length of fibers in untreated NEIL3 proficient (n=99) and NEIL3 deficient cells (n=129) versus HU treated NEIL3 proficient (n=110) and NEIL3 deficient cells (n=120); H_2_O_2_ treated proficient (n=207) and deficient (n= 207); Cisplatin treated NEIL3 proficient (n=207) and deficient (n=245); **(J)** Distribution of fiber length in NEIL3 proficient versus deficient cells with or without HU, H2O2 and cisplatin. All data were analyzed using GraphPad Prism software.

To determine whether NEIL3 is required to protect cells from oxidative and replication stress-induced DBSs, we used γ-H2AX and 53BP1 (tumor protein 53 binding protein No. 1) nuclear foci to monitor DSBs. Our data show that the number of γH2AX co-localized with 53BP1 were significantly increased in NEIL3 deficient versus NEIL3 proficient cells treated with HU (Figure 2A-2B; 75± 4 versus 45±5; P= 0.0001). Furthermore, DSBs significantly increased in NEIL3 deficient versus proficient cells after treatment with H_2_O_2_ (Figure 2A-2B, 57±5 versus 38±4; P=0.0001). Interestingly, our data show that levels of spontaneous DSBs were significantly increased in the NEIL3 deficient cells versus proficient cells (Figure 2A-2B; 16 ± 3 versus 5±2; P=0.0001). Furthermore, using a neutral comet assay we reconfirmed that DSBs were significantly increased in NEIL3 deficient LN428 and LN18 cells treated with HU (P=0.002 versus P=0.02) or H_2_O_2_ (P=0.0001 versus p=0.004) compared to NEIL3 proficient cells respectively (Figure 2C and 2D). In order to determine whether the DSBs are associated with actively replicating DNA, we performed co-immunostaining of the cells with antisera against CIdU and γH2AX as a marker of replication stress [29]. NEIL3 proficient and deficient cells were pulse-labeled with CIdU for 30 min and treated with HU (2mM) for 2 hours (Figure 3A). The number of double-positive cells (CIdU+ γH2AX) was significantly higher in NEIL3 deficient cells than in NEIL3 proficient cells (Figure 3B, 62 ± 5.8; P=0.0001). To determine whether the loss of NEIL3 impairs DNA synthesis after replication stress, we evaluated the co-localization of IdU and CIdU. Our result show that co-localization of IdU and CIdU were significantly decreased in NEIL3 deficient versus proficient cells (Figure 3C and 3D; 6 ± 2; P=0.0001), suggesting that NEIL3 may be required to maintain replication fork integrity after replication stress. Moreover, to determine if NEIL3 protects the cells from oxidative or replication stress-associated cytotoxicity, we conducted clonogenic cell survival assays to compare NEIL3-deficient versus proficient cells. We treated cells with different concentrations of H_2_O_2_ (100nM, 200nM, 400nM) for 4 hours or with HU (1, 2,3, 4 and 6mM) for 2 hours. The percent of surviving cells significantly decreased in NEIL3 deficient cells treated with H_2_O_2_ or HU compared to NEIL3 proficient cells (Figure 2F P=0.0001 and Figure 2G; P=0.0001). Interestingly, exogenous expression of NEIL3 complements NEIL3 deficient cells and decreases the sensitivity to HU or H_2_O_2_ treatment (Figure 2F-2G).

### Loss of NEIL3 inhibits DNA replication fork progression

To determine if NEIL3 is required for replication fork progression, we used DNA fiber labeling methods to measure the progression of replication forks [30]. Replication tracts in NEIL3 proficient and deficient cells were first labeled with IdU (25μM) for 30 minutes, then treated with three different DNA damaging agents (200nM of H_2_O_2_ for 1 hour; 2 mM HU for 2 hours; 1 μM cisplatin for 1 hour) followed by a second labeling with CIdU (250μM) for 30 minutes as described in Figure 3E. Interestingly, our data show that NEIL3 deficient cells spontaneously exhibited 36% stalled forks, which increased significantly up to 59% after two hours of HU as compared to HU-treated NEIL3 proficient cells (50%) (Figure 3F, P=0.0001). Similarly, the percentage of stalled replication forks significantly increased in H_2_O_2_-treated NEIL3 deficient cells (57%) versus treated NEIL3 proficient cells (42%) (P=0.0001). In addition, cisplatin treatment in NEIL3 deficient cells resulted in significantly more stalled forks than cisplatin treatment in proficient cells (Figure 3F; 71% versus 52%; P=0.001). Moreover, the speed of the replication forks was significantly reduced to 0.3kb/min in HU treated NEIL3 deficient cells compared to 0.7kb/min in NEIL3 proficient cells (Figure 3G; P=0.0001). Similarly, the average replication speed was significantly reduced to 0.26kb/min and 0.04 kb/min in H_2_O_2_ and cisplatin treated cells respectively. Together, these data suggest that NEIL3 is required for replication fork progression both intrinsically and after oxidative DNA damage or replication stress.

### Oxidative and replication stress-associated DNA damage exacerbate the instability of nascent DNA strands in NEIL3 deficient cells

To determine whether oxidative and replication stress-associated DSBs induce nascent DNA strand instability in NEIL3 deficient cells, the NEIL3 proficient and deficient cells were pulsed with IdU for 30 minutes and treated with DNA damaging agents (2mM HU; 1 hour of 200 nM H_2_O_2_ and 1μM cisplatin) (Figure 3H). We noticed that DNA fibers that contained IdU tracts were significantly shorter in HU, H_2_O_2_ or cisplatin-treated NEIL3 deficient cells versus NEIL3 proficient cells (Figure 3H-3I; Mean+ SEM; 8.2 μm ±0.3; 1.9 μm ± 0.08; 1μm ± 0.05; versus 6.7 μm ±0.24; 2.7 μm±0.24; 1.5 μm ±0.11, P=0.0001). Twenty percent of DNA fibers in NEIL3 deficient cells had lengths that were less than or equal to 5μm versus wild type (6%), implying that the collapsed replication forks were not maintained in NEIL3 deficient cells(Figure 3G; P=0.001). Furthermore, treatment with HU, H_2_O_2_ and cisplatin significantly increased the relative frequency of fiber length less than or equal to 5μm in NEIL3 deficient cells to 45% and 98% to 98% respectively (Figure 3G; P=0.0001).

### NEIL3 is associated with replication forks, and its deficiency impairs DSB repair

**Figure 4:**
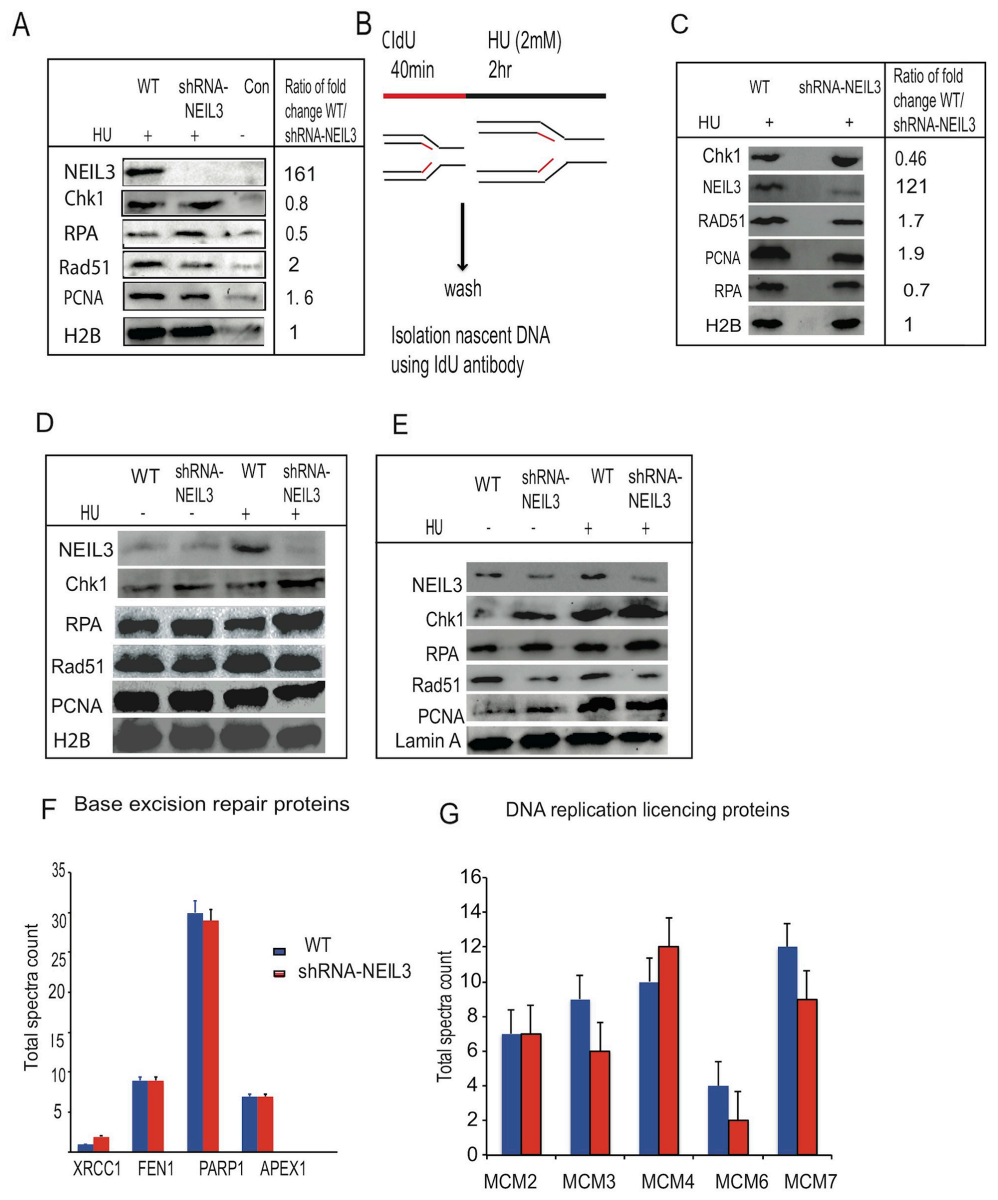
NEIL3 is associated at replication fork during replication stress and its loss impaired the recruitment of replication associated homologous repair proteins **(A)** The recruitment of NEIL3, Rad51, RPA, PCNA, and CHK1 after HU treatment at new synthesized DNA fragments; **(B)** Schematic representation of replication fork recovery and the recruitment of homologous recombination proteins at the newly synthesized DNA after replication stress (2mM HU); **(C)** Protein bound to immunoprecipitation (ChIP) with IdU antibody after replication stress; **(D)** Chromatin associated fractions; **(E)** Nuclear fraction; **(F)** Base excision repair proteins recruited at newly synthesized DNA after replication stress in NEIL3 proficient and deficient cells; **(G)** Replication licensing proteins recruitment in NEIL3 proficient and deficient cells. No statistically significant difference was observed between NEIL3 proficient and deficient cells in data F and G.

To determine whether NEIL3 is associated with the newly synthesized DNA fragment at the replication fork, we performed an applied isolation of proteins on nascent DNA (IPOND) assay as previously described [31, 32]. Our results revealed the presence of NEIL3 in newly synthesized DNA fragments in HU treated cells, suggesting that NEIL3 is bound to DNA in order to overcome stalled replication forks (Figure 4A-4C). We did not detect NEIL3 signal with control sample (no click chemistry reaction) and shRNA-NEIL3 knockdown cells, which further confirmed the specificity of the NEIL3 band (Figure 4A). Further, to reconfirmed whether NEIL3 is involved in the recruitment of HR proteins during replication fork recovery after DNA damage, we conducted immunopreciptation experiments on newly synthesized DNA strands after pulsing the cells with chlorodeoxyuridine (CldU) (Figure 4B). We found that NEIL3, RPA, RAD51, Chk1 and PCNA were associated with newly-replicated DNA strands (Figure 4C). As expected, the levels of DNA-associated RAD51 and PCNA at the replication fork were significantly reduced in NEIL3 deficient versus proficient cells in the HU treated group (Figure 4C). To examine whether NEIL3 is associated with chromatin during replication stress, we performed a fractionation procedure to examine replication factors in different cellular fractions (Figure 4D-4E). We found that chromatin-associated Rad51, PCNA and Chk1 are moderately reduced in HU- treated NEIL3-deficient cells compared to proficient cells (Figure 4D). Furthermore, high levels of RPA, PCNA and RAD51 were detected in nuclear extracts of NEIL3 deficient versus proficient cells (Figure 4E). Further, our IPOND-mass spectrometry data show that no statistically significant difference exists between base excision repair proteins (APEX1, FEN1 and XRCC1, Figure 4F) and DNA replication licensing proteins (MCM 2-7) in NEIL3 deficient versus proficient cells (Figure 4G). However, our results show that six PCNA interacting proteins (CCP110, MSH6, BAZ1B, SMARCA5, SMARCA1 and DNMT1) were significantly decreased in the absence of NEIL3 (Supplementary Figure 1A; P=0.013 and Supplementary Table 1). In addition, the recruitment of Topoisomerase I and Topoisomerase 2A on nascent DNA at the replication forks was significantly decreased in NEIL3 deficient cells versus proficient cells (Supplementary Figure 1B; P=0.0075; P=0.04). All the peptide sequence were collected from mass spectrometry data were analyzed using the scaffold viewer application soft ware (Supplementary Table 1).

### NEIL3 deficiency decreases Rad51 recruitment but not RPA

**Figure 5:**
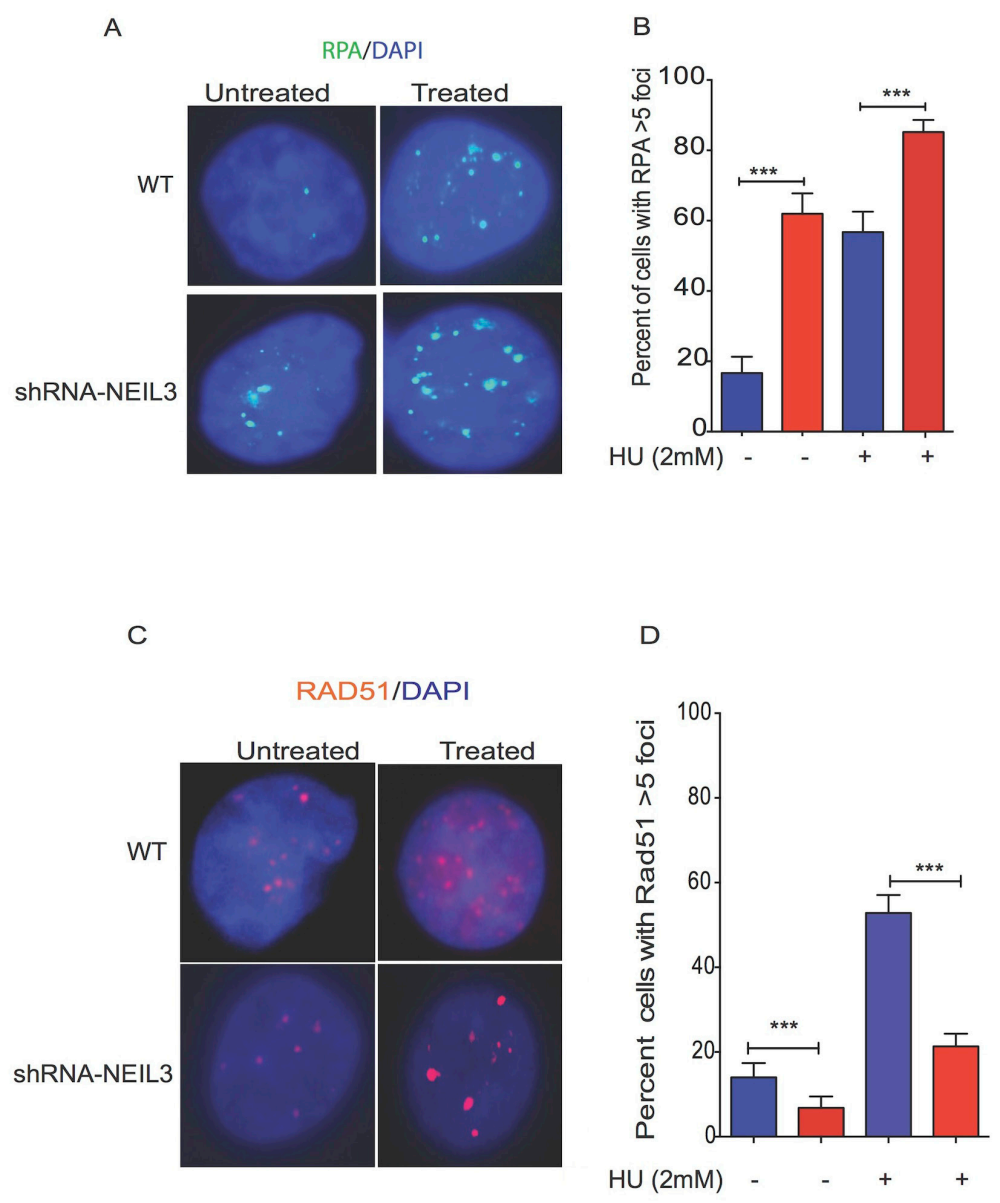
Replication stress alters the localization of Rad51 and RPA in NEIL3 deficient versus proficient cells Subcellular localization of RAD51 and RPA in NEIL3 proficient versus deficient cells; **(A)** Representative images of RPA localization in NEIL3 deficient and NEIL3 proficient cells; **(B)** Quantification of percent of cells with RPA greater than 5 foci per nuclei. Note that the number of untreated NEIL3 proficient cells (n=66) and deficient cells (n=71) versus treated proficient (n=74) and deficient cells (n=108) was included for analysis. **(C)** Rad51 localization in NEIL3 deficient and NEIL3 proficient cells before and after HU treatment; **(D)** Estimated percent of positive cells for Rad51 foci. Cells with at least 5 foci were counted as Rad51 positive cells and analyzed with GraphPad prism software. Note that the number of untreated NEIL3 proficient (n=108) and NEIL3 deficient cells (n=88) versus treated NEIL3 proficient (n=140) and NEL3 deficient cells (n=192) was included in this analysis.

To determine the mechanism of NEIL3 replication-associated DSBs repair, we examined the localization of RPA and RAD51 in NEIL3 deficient versus proficient cells before and after replication stress (Figure 5A). The percentages of RPA positive cells were increased in NEIL3-deficient cells, suggesting that ssDNA levels were increased in the absence of NEIL3 (Figure 5B, P=0.001). To investigate the HR response, cells were treated for 2 hours with HU (2mM) and stained for the recombination protein RAD51 (Figure 5C). As expected HU exposure efficiently induced RAD51 foci in NEIL3 proficient cells (Figure 5D). However, the numbers of cells with Rad51 foci (>5 foci) were significantly decreased in NEIL3-deficient cells (Figure 5D; P<0.001).

### NEIL3 deficient cells are sensitive to ATR and PARP1 inhibitor

**Figure 6:**
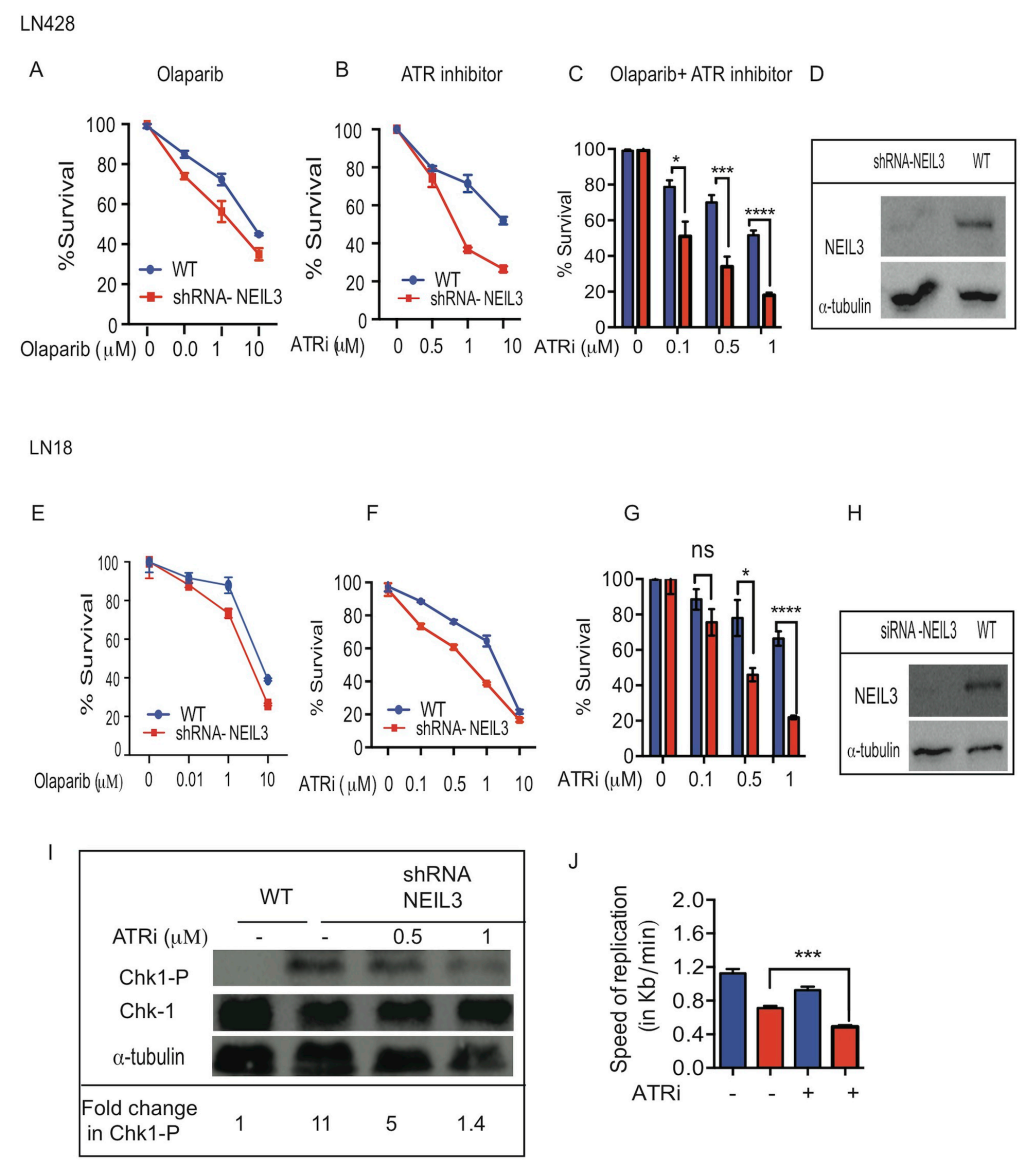
Loss of NEIL3 confers sensitivity to ATR inhibition and synergizes with PARP1 inhibitor Estimated percent of clonogenic survival of NEIL3 proficient versus NEIL3 deficient cells with different concentration of Olaparib, ATR inhibitor and in combination (ATR inhibitor plus Olaparib (10nM)). **(A-C)** LN428 treated with Olaparib only (B) ATR inhibitor only (C) combination of ATR inhibitor and olaparib; **(D)** Western blot for knockdown of NEIL3 in LN428 cells; **(E-G)** LN18 NEIL3 proficient versus deficient cells treated with Olaparib; **(H)** Western blot for NEIL3 knockdown in LN18 cells **(I)**; Western blot for Chk1 phosphorylation (Ser317) and Chk1 after ATR inhibition. Note that the ratio of the Chk1 (Ser317) versus total Chk1 signals for each lane are provided as an estimate of the relative stoichiometry of Chk1 Ser317 phosphorylation in each fraction; **(J)** Replication speed before and after ATR inhibitor in LN428 cells.

PARP1 is associated with replication forks [33] and its inhibition leads to replication fork associated DSBs [34]. To assess cellular toxicity to a single or combined treatment of PARP1 inhibitor (Olaparib; 10nM, 1μM and 10μM) and/or ATR inhibitor (AZD6738; 0.5μM; 1μM, 10μM and 20μM), we performed clonogenic survival assays using two different glioblastoma cell lines (LN428 and LN18). First, we determined that loss of NEIL3 increased the cytotoxicity response to PARP1 inhibitor. Our data show that the percentage of cell survival is significantly decreased in NEIL3 deficient versus proficient cells treated at 1μM and 10 μM (Figure 6A and 6D; P= 0.001). Moreover, our data show that ATR inhibitor (0.5μM; 1μM; 10μM) significantly increased sensitivity in NEIL3 deficient versus proficient cells (Figure 6B and 6E; P=0.002; P=0.0023). In contrast, a high concentration of ATR inhibitor (20μM) caused cell lethality in both wild type and NEIL3 deficient cells (Supplementary Figure 2). To examine the synergistic cytotoxicity response of the two drugs in NEIL3 deficient cells, we treated cells with ATR and PARP1 inhibitors and calculated the combination index (CI) using Calcusyn software (Biosoft) based on the method described by Chou et al [35]. Our data show that PARP1 inhibition (10nM and 1μM) synergized with ATR inhibitor in LN 18 and LN428 NEIL3 deficient gliobastoma cell lines. The CI<1 (0.2 (LN18); and 0.09 (LN428)), suggested that the synergistic effects in NEIL3 deficient cells are greater when the combination of agents is used (Figure 6C and 6F; P=0.0001).

## DISCUSSION

DNA glycosylases are mainly known for initiating the BER pathway that protects cells from the mutagenic and/or cytotoxic effects of DNA base lesions [2, 36, 37]. The NEIL DNA glycosylases recognize and remove a vast number of oxidized base lesions, with overlapping substrate specificity [12, 13, 38, 39]. NEIL3 functions in the BER pathway to catalyze the excision of DNA bases that have been damaged by ionizing radiation or other sources of reactive oxygen species [4, 40, 41]. NEIL3 is overexpressed in several cancers including glioblastoma multiforme (GBM) and significantly reduces survival rates [42]. In this study, we addressed whether NEIL3 is required to overcome oxidative and replication-associated DNA damage and examined the therapeutic advantage of aberrant NEIL3 in cancer cells.

Therefore, we investigated the role of NEIL3 in DNA replication and repair in more detail. First, we found that NEIL3 is localized to DSB sites, suggesting that NEIL3 is likely participates in oxidative or replication stress-induced DNA damage repair. Our results are consistent with the notion that BER proteins are involved in replication-associated DNA damage repair [36, 43]. We have further found that NEIL3 co-localized with replicative DNA foci (CIdU), suggesting that NEIL3 is likely involved in maintenance of replication fork integrity (Figure 1A). The cell cycle dependent expression of NEIL3 was reported in the S phase and continues into the G2 phases [16, 44, 45], which is consistent with our results.

Several studies have shown that replication-associated DNA damage represents a major challenge to genomic integrity [46], as illustrated by the numerous proteins required for DNA replication progression and recovery [47]. Interestingly, our data demonstrated that NEIL3-deficient cells encountered a significant increase in spontaneous replication stresses that challenged replication fork progression and provoked replication fork collapse (Figure 2A). The co-localization of γH2AX foci with replicative DNA foci in NEIL3 deficient cells suggests that DSBs occur during DNA synthesis in the presence of oxidative and replication-associated DNA damage (Figure 3A) [48–50]. We propose two alternative explanations, not necessarily mutually exclusive, for how NEIL3 deficient cells are sensitive to HU or H_2_O_2_ treatment. The first possibility is that the pre-existing genomic instability or the exhaustion of essential DNA repair factors fails to repair the Sp and Gh lesions and renders NEIL3-deficient cells more vulnerable to exogenous genotoxic stress resulting in SSBs [51, 52], and those unrepaired SSBs are likely to give rise to highly cytotoxic DSBs and chromosomal instability [53]. Alternatively, DSBs generated by oxidative stress (H_2_O_2_) can also provoke replication fork collapse (Figure 3). Moreover, oxidative stress can affect replication through additional processes, such as by regulating the activities of replication proteins [54] [21–26]. The second scenario we propose is that NEIL3 deficient cells may fail to recruit chromatin remodeling protein complexes (Supplementary Figure 1) or resolve the topological constraint during replication [55–57] to prevent the replicative DNA polymerase from stalling at the replication fork [58, 59]. Our results provide evidence that NEIL3 likely maintains replication-associated chromatin structure to recruit DNA repair proteins [60]. Even if NEIL1 interacts with replication proteins and mediates pre-replicative repair of oxidized bases in the replicating strand [61], our data suggest that NEIL3 loss is unable to be restored by NEIL1 repair proteins. Altogether, it is plausible to suggest that NEIL3 and NEIL1 DNA glycosylases have evolved to carry out different specialized functions that do not overlap in our experimental model.

Many HR proteins form nuclear complexes and are recruited at DNA damage sites [62]. The ssDNA template at the replication fork may be more prone to oxidative DNA damage and strand breaks than non-replicating DNA, thus warranting its urgent repair to prevent genomic instability. Replication stress leads to exposure of tracts of ssDNA that are substrates for endonuclease cleavage, which generates DSBs [46]. Interestingly, our data showed that RPA is more efficiently recruited in NEIL3 deficient cells, which is a key factor to stabilize ssDNA [63], suggesting that collapsed replication forks likely generate long stretches of ssDNA and trigger a replication stress response [64, 65]. Based on the studies of Nam et al, it appears that a stalled replication fork causes uncoupling of the replicative DNA polymerase/helicase complex, which leads to persistent exposure of ssDNA that is subsequently bound by RPA [66]. It is possible that the persistence of ssDNA in NEIL3 deficient cells likely exhausts the nuclear pool of RPA in NEIL3 deficient cells, and accelerates replication-associated DSBs [67]. However, we did not observe a significant difference in replication licensing proteins (MCM) complex recruitment at the replication fork in NEIL3 deficient versus proficient cells, suggesting that the loss of NEIL3 does not promote unscheduled new origin firing. Alternatively, excess RPA recruitment in NEIL3 deficient cells likely triggers the ataxia-telangiectasia-and Rad3-related (ATR) protein kinase signaling pathway to regulate replication stress [68, 69]. Our data showed that NEIL3 deficient cells spontaneously activated Chk-1 phosphorylation [317] and decreased the speed of replication (Figure 6G-6H). ATR is a key player in DNA damage response to stabilize or repair DNA replication fork collapse during the S-phase [67, 70–73]. We demonstrated that ATR inhibitor increased stalled replication forks in NEIL3 deficient cells and attenuates Chk-1 activation suggesting that ATR/Chk-1 signaling is critical to protect replication fork integrity in NEIL3 deficient cells. Our observation is consistent with previously published data suggesting that ATR and its downstream effectors significantly counteract the adverse effects of replication stress both by delaying cell-cycle progression and stabilizing stalled forks [74]. Together, our data suggest that the inhibition of ATR in NEIL3 deficient cells significantly impairs replication fork progression.

Several studies have shown that Rad51 recruitment to replication forks is a prerequisite for replication fork recovery [75, 76]. Our data show that NEIL3 proficient cells exhibit Rad51 recruitment and prevent replication-associated DSBs at the replication fork (Figure 4), similar to previously published data [77, 78]. In contrast, we have observed markedly decreased Rad51 recruitment, suggesting that HR dependent repair is compromised in NEIL3-deficient cells (Figure 5C). Our data led us to offer two alternative possibilities. First, the unstable nascent DNA strand near the replication fork in NEIL3 deficient cells probably contributes to less Rad51 protein recruitment (Figure 3B). Second, the excess presence of RPA on ssDNA may prevent Rad51 foci formation or binding on newly synthesized DNA strands in NEIL3 deficient cells, suggesting that RPA-ssDNA formation suppresses Rad51 presynaptic filament formation [79–81]. Nevertheless, these observations are consistent with other *in-vitro* published data that have shown critical interactions between RPA and Rad51 [79, 80, 82].

NEIL3 deficient cells are prone to oxidative DNA base damage, abasic sites, and strand breaks that likely offer an additional avenue to increased sensitivity in cancer cells. Our studies demonstrated that HU-mediated dNTP pool depletion significantly increased cytotoxicity in NEIL3 deficient cancer cells, suggesting that the NEIL3 status of tumors should be routinely assessed to improve treatment response. It is possible that HU treatment induced replication stress-mediated oxidative DNA damage in NEIL3 deficient cells. Previously, Kang et al shows that HU treatment increases intracellular ROS levels that promotes genomic toxicity [83]. Moreover, we showed that defects in replication fork progression (Figure 2A, Figure 3) increased the cytotoxic effect of Olaparib in NEIL3 deficient cells. Our observation is in agreement with other studies that indicate PARP1 is associated with replication forks [33] and inhibition of PARP1 leads to stalled replication fork and induces DSBs [34]. Alternatively, cancer cells treated with Olaparib would create a need for ATR activity that likely promotes cell cycle check-point activation and DNA repair [84]. Previous data reported that combination of ATR and chemotherapy significantly increased sensitivity in cancer cells [85, 86]. In addition, cells lacking BER scaffold protein, XRCC1, are hypersensitive to ATR inhibitor-induced cytotoxicity [87]. In line with these observations, we confirm that NEIL3 deficient cells are sensitive to ATR inhibitor. Interestingly, the combination of ATR inhibitor with Olaparib synergistically sensitizes NEIL3 deficient cancer cells (Figure 6A-6F), suggesting that blocking the ATR-mediated DNA damage response synergizes the cytotoxicity of PARP1 inhibitor.

In conclusion, our data indicate that the alteration in NEIL3 function in cancer cells likely drives replication-associated genomic instability. This suggests that an ATR inhibitor as a single therapy or in combination with a PARP1 inhibitor is likely effective to treat NEIL3 deficient tumors. Our findings may have important clinical implications for the use of an ATR inhibitor to synergize the cytotoxicity of a PARP1 inhibitor in patients who harbor NEIL3-deficient tumors. However, it is critical to examine the genetic status of a given tumor for NEIL3 deletion, amplification or mutation to determine a better treatment outcome. Together, these studies suggest that conditions of increased ROS levels, as well as defective BER, may provide contexts in which GBM patients might be amenable to a single PARP1 inhibitor or combination therapeutic strategies that are likely useful for the future. Further, our results offer testable, predictive alternative approaches based on the NEIL3 status of tumors and may inspire further work to validate how NEIL3 overexpression promotes resistance to replication stress and also to determine whether NEIL3 glycosylase activity is required for the maintenance of the replication fork integrity.

## MATERIALS AND METHODS

### Cell lines and culture conditions

The LN428 glioblastoma cell lines were obtained from Trevigen. Both the wild-type (Catalog Number 5503-001-01) and the knocked-down, NEIL3-deficient experimental cells (Catalog Number 5508-001-01) were maintained in MEM-α supplemented with 10% Fetal Bovine Serum (FBS), 1% Penicillin/ Streptomycin (P/S), 1% L-Glutamine and 1μg/mL Puromycin at 37°C in a cell culture incubator maintained at a 5% CO_2_ level in a humid environment. The media was supplemented with fresh puromycin every three days. The LN18 cell line was obtained from ATCC (Catalog Number CCL-185). These cells were grown in DMEM supplemented with 10% FBS and 1% P/S at 37°C in a cell culture incubator maintained at a 5% CO_2_ level in a humid environment.

### Transfection, infection, and expression analysis

NEIL3-deficient LN428 cells were seeded at 20,000 cells per cover slip in culture media for 24 hours. Cells were then transfected with an N-terminally tagged GFP-NEIL3 plasmid (Origene, Catalogue Number RG206838) using Lipofectamine 2000

(ThermoFisher, Catalogue Number 11668027) as per the manufacture instructions. Cells were cultured for 24 hours, washed with PBS, and then used for further applications outlined below. Human NEIL3 constructs were packaged into a retrovirus using the GP2-293 packaging cell line. pRVY-Tet and pVSV-G plasmids were co-transfected into GP2-293 cells using standard calcium phosphate transfection. Approximately 30% confluence of LN428 cells were infected with retrovirus in the presence of 4 μg/ml polybrene. For selection of pools, cells were split 1:3 the day after infection and cells with the integrated construct were selected with 200μg/ml hygromycin B. Expression of exogenous HA tagged NEIL3 was verified by western blot. Cells were passed in parallel in the presence or absence of tetracycline. Approximately 80-90% confluent cells were harvested by scraping with hot SDS Loading Buffer (50 mM Tris pH 6.8, 100 mM DTT, 2% SDS 10% glycerol). Lysates were boiled for 10 minutes and run on a 10% acrylamide SDS-PAGE gel. Proteins were transferred to nitrocellulose membrane using a semi-dry transfer apparatus and probed using monoclonal mouse anti-NEIL3 antibody (abcam #1831).

### Small Interfering RNA (siRNA) for knockdown of NEIL3

For the siRNA studies, the 21-mers of the siRNA duplexes directed against NEIL3 with were used to select siGenome ON-TARGETplus SMART pool (Catalog number L02093 9-01-0005) and were synthesized by Dharmaco Research Inc. (Lafayette, CO). All siRNA was resuspended in RNase – free water for a final concentration of 20 μM, and the siRNA was aliquoted into small volumes and stored at −80°C until the experiment was performed. All RNAi sequences are :-J 020939-09: 5’GCU AAU GGA UCA GAA CGU3’; J-020939-10: UAA UGA AGU ACC CGU GUA AA 3’; J-020939-11: CUA UGU AUU UCA UCG GAU A 3’; J-020939-12: AGAAGA CAA CAA ACG AUA U-3’).

### Drug treatment of cell culture

LN18 and LN428 cells were treated with a variety of drugs referred to throughout the paper by first thawing a fresh aliquot of concentrated stock. Hydroxyurea (Sigma, Catalogue Number H8627) was applied to cells at a final concentration of 2 mM for 1.5 hours in culture. ATR Inhibitor (Selleck, Catalogue Number AZD6738) was applied to cells at a range of concentration of 0.5-20μM for 2 hours in culture. Hydrogen Peroxide (Sigma, Catalogue Number H3410) was applied to cells at a final concentration of 1 μM for 1 hour in culture. Cells were then washed with PBS and used for the applications outlined below.

### Immunofluorescence localization

Cover slips were seeded with 20,000 cells and cultured for 24 hours. Any drug treatments or transfections were appropriately performed (see above). Then, cells were fixed with 3.5% formaldehyde or methanol:acetic acid (3:1 ratio) for 10 minutes. Cells were then permeabilized in PBS containing 0.5% Triton X-100 for 15 minutes at room temperature. Then, cells were blocked with 3% BSA in PBS for 1.5 hours at room temperature. Primary antibody was diluted to a 1:100 concentration in blocking buffer and incubated with cells overnight at 4°C. Primary antibodies include mouse anti-GFP antibody (Origene, Catalogue Number 150041), rabbit anti-RAD51 antibody (SantaCruz, Catalogue Number sc-8349), mouse anti-RPA antibody (Abcam, Catalogue Number ab2175), mouse Anti-phospho-Histone H2AX (Millipore, Catalogue Number 05-636I), rabbit 53BP1 (SantaCruz, Catalogue Number sc-22760) and rabbit anti-γH2AX antibody (Bethyl, Catalogue Number A300-081A). On the following day, cells were washed with PBS and then incubated with secondary antibody diluted to a 1:400 concentration in blocking buffer for 1.5 hours. Secondary antibodies include FiTC conjugated anti-mouse antibody (Jackson Labs, Catalogue Number 715-095-150), Texas Red anti-goat antibody (Life Technologies, Catalogue Number PA1-28662) and TRITC conjugated anti-rabbit antibody (Jackson Labs, Catalogue Number 111-295-045). Finally, cells were washed with PBS and mounted with cover slips using mounting media containing DAPI stain. After the basic primary and secondary antibody staining outlined above, DNA synthesis was detected using antibody against BrdU by further processing the cells. Cells were washed twice with PBS and then fixed again with methanol: acetic acid (3:1 ratio) for 5 min. Cells were then incubated for 10 min at 37°C in 1.5 N HCl. Cells were washed with PBS and then incubated at RT in 0.5% Tween for 5 min. To block the samples, cells were incubated at RT in 3% BSA for 20 min prior to antibody addition. Primary and secondary antibodies at this stage were added as above, using primary mouse anti-BrdU antibody (BD Labs, Catalogue Number 347580) and secondary TRITC anti-mouse antibody (Jackson Labs, Catalogue Number 115-295-003).

### Clonogenic survival assay

Control cells and NEIL3-deficient cells were plated at 500 cells per well in six-well plates and cultured to adhere overnight. Cells were then treated with a single agent for 24 hours: Olaparib (10nM, 1 μM, 10 μM), or ATR inhibitor (0.5 μM, 1 μM, 1.5 μM, 10 μM, and 20 μM). Cells treated with 10-20 μM ATR inhibitor were additionally repeated and plated at 1,000 cells per well to acquire a more statistically relevant cell survival curve. Additionally, cell survival was recorded when treated in combination with ATR inhibitor and Olaparib. Control cells and NEIL3-deficient cells were plated at 1,000 cells per well in six-well plates and cultured to adhere overnight. Cells in the Olaparib group were treated with Olaparib and ATR inhibitor concurrently for 24 hours. For H_2_O_2_ and HU treatment, cells were treated with H_2_O_2_ (50 nM, 100 nM, 200 nM and 400 nM) for 4 hours. Following treatment, cells were washed with PBS and supplemented with fresh growth media. The cells were cultured for 10 days before staining with 0.25% crystal violet in an 80% methanol solution. Plates were allowed to dry, and then colonies were counted and scored visually. All experiments were repeated twice to improve statistical significance.

### Single-cell gel electrophoresis (neutral comet assay)

Equal numbers of cells (1x 10^5^) were plated in 10cm dishes. The day after, cells were treated with 2mM HU or 1000 nM H_2_O_2_ for 1 hour. After treatment, the cells were prepared load on Trevigen CometSlide™ (Catalogue number 4250-050-03) and slides immersed in lysis buffer (Catalogue number: 4250-050-01). The slides were subjected to electrophoresis using neutral electrophoresis buffer and further processed according to published procedures (Trevigen, Catalog #4250-050-K). The slides stained with SYBR Gold and visualized using Fluorescein filter of Carl Ziess microscope with Axiocam MRc5 color camera. Image analysis of 100 cells was performed using Comet Assay IV software (Instem, Conshohocken, PA). Data are represented as means ± SEM.

### DNA fiber analysis

For DNA replication analysis, sequential labeling of DNA with IdU and CldU were performed based on previously described methods [57]. A sub-confluent, asynchronous population of NEIL3 proficient and deficient LN428 cells was first labeled for 30 min with 25μM IdU, then washed with medium three times. After the CIdU pulse, three sets of experiments were conducted and cells were treated with three different agents (2mM HU for 2 hr; 200nM H_2_O_2_ for 1 hour; 1μM cisplatin (CTP) for 1 hour). The cells were then labeled for another 30 min with 250μM CldU. After incubation, cells were washed and resuspended at a concentration of 7.5×10^5^ cells/ml. The number of cells lysed per slide ranged between 1500 to 5000 cells using fiber lysis buffer (50mM EDTA, 0.5% SDS, 200mM Tris-HCl, pH=7.5) for 2 minutes, and the slides were tilted at 20° for gravity flow. The untreated control cells used were pulsed for 30 minutes with IdU, followed by 1 hour with media only, then pulsed with CIdU label for 30 minutes, and the cells were harvested for the fiber assay. For immunofluorescence staining, the slides were fixed for 10 minutes with methanol: acetic acid (3:1) and air-dried. The slides were treated with 2.5M HCl for 30 minutes, washed with 1xPBS three times, and then blocked with 3% BSA/PBS for 1 hour. CldU was detected by incubating acid-treated fiber spreads with rat anti-BrdU monoclonal antibody (Abcam), and IdU was detected using mouse anti-BrdU monoclonal antibody (1:1,000; Becton Dickinson) for 1 hr at room temperature. This was followed by washing three times with 1x PBS and stained with secondary antibody conjugated with sheep anti-mouse Cy3 and goat anti-rat Alexa fluor 488 for 1 hour at room temperature. The slides were mounted with Vectashield mounting media and covered with coverslips. Images were acquired at 63x magnification using a Zeiss microscope and processed and analyzed using the ImageJ program. The lengths of red (Cy3) or green (AF 488) labeled patches were measured using the ImageJ software (National Institutes of Health; http://rsbweb.nih.gov/ij/) and arbitrary length values were converted into micrometers using the scale bars created by the microscope. Data analysis was carried on using the ImageJ software. We applied the conversion factor 1 μm = 2.59 kb [88].

### CldU coimmunoprecipitation of proteins at stalled replication forks

NEIL3 proficient and deficient (LN428) cells (2 × 10^6^) were plated and after 24 hours labeled with 100 micromolar CIdU for 40 min and cells were treated with 2mM HU for 2 hours. Cells were cross-linked in 1% PFA for 15 min. The cytoplasmic protein fraction was removed by incubation in hypotonic buffer (10 mM HEPES [pH 7], 50 mM NaCl, 0.3 M sucrose, 0.5% TX-100, and protease inhibitor cocktail) for 10 min on ice followed by centrifugation at 1500 g for 5 min. Nuclear soluble fraction was removed by incubation with nuclear buffer (10 mM HEPES [pH 7], 200 mM NaCl, 1 mM EDTA, 0.5% NP-40, and protease inhibitor cocktail) for 10 min on ice and centrifugation at 13,000 rpm for 2 min. Pellets were resuspended in lysis buffer (10 mM HEPES [pH 7], 500 mM NaCl, 1 mM EDTA, 1% NP-40, and protease inhibitor cocktail), sonicated, centrifuged for 30 s at 13,000 rpm, and the supernatant was subsequently transferred to a new tube. Total protein (150 μg) was used for IP with 2 μg anti-CldU antibody (rat-anti-BrdU; OBT0030F AbD Serotec) and 20μl of Protein A/G-PLUS agarose overnight at 4°C (Santa Cruz Biotechnology). The IP reaction was washed twice with nuclear buffer and twice with washing buffer (10 mM HEPES and 0.1mM EDTA protease inhibitor cocktail), incubated in 2× sample loading buffer (100 mM Tris HCl [pH 6.8], 100 mM DTT, 4% SDS, 0.2% bromophenol blue, and 20% glycerol) for 30 min at 90°C, and was used for Western Blot with mouse anti-Rad51 (Millipore (Catalogue Number 05-530) and mouse ant-γH2AX (Millipore, Catalogue Number 05-636) primary antibodies at a dilution of 1:1000 in 3% non-fat milk in PBST.

### IPOND (Isolation of Proteins on Nascent DNA)

Protein association to nascent DNA was measured by following the iPOND Nature Protocol outlined by Sirbu et al [32]. Briefly, WT or NEIL3-deficient LN428 cells were cultured as outlined above in 75cm^2^ culture flasks in 10 mL of complete media until 50% confluent and 1×10^8^ cells total. Cells were first pulsed with 10 μM EdU (Sigma, Catalog Number T551285-5MG) for 30 min., washed, and then treated with or without 2 mM HU as outlined above for 2 hrs. Cells were immediately fixed with 1% formaldehyde at RT for 20 min., quenched with 1.25 M glycine, and collected by scraping into a 50 mL conical tube on ice. Cells were then pelleted at 4°C and washed three times with PBS, permeabilized, washed with 0.5% BSA in PBS, and treated with a click reaction cocktail containing biotin azide (Invitrogen, Catalog Number B10184). Biotin-bound nascent DNA was then captured with streptavidin beads (ThermoFisher, Catalog Number S951), and associated proteins were collected and analyzed via Western blot or mass spectrometry. We used the rabbit anti-chk-1 (Ser317) (Cell Signaling; Catalogue Number 2344s); mouse anti-RPA antibody (Abcam, Catalogue Number ab2175); mouse anti-Rad51 (Millipore (Catalogue Number 05-530); PCNA (Santa Cruz; Catalogue Number SC-56); H2B (Abcam, Catalogue Number ab1790.); Rabbit anti-NEIL3 (AbCam Catalogue Number ab174205) antibodies.

### Statistical analysis

All reported data was evaluated in a pairwise comparison, including NEIL3-proficient versus deficient cells using GraphPad Prism.

## SUPPLEMENTARY MATERIALS FIGURE AND TABLE




