# Supplementary material for: Loss of NEIL3 DNA glycosylase markedly increases replication associated double strand breaks and enhances sensitivity to ATR inhibitor in glioblastoma cells
Source: Oncotarget. 2017 Dec 4;8(68):112942–58. doi: 10.18632/oncotarget.22896 (PMC5762564; doi:10.18632/oncotarget.22896)
Supplement: Supplementary file 1 [file oncotarget-08-112942-s001.pdf]

Loss of NEIL3 DNA glycosylase markedly increases replication associated double strand breaks and enhance sensitivity to ATR inhibition in glioblastoma cells

SUPPLEMENTARY MATERIALS

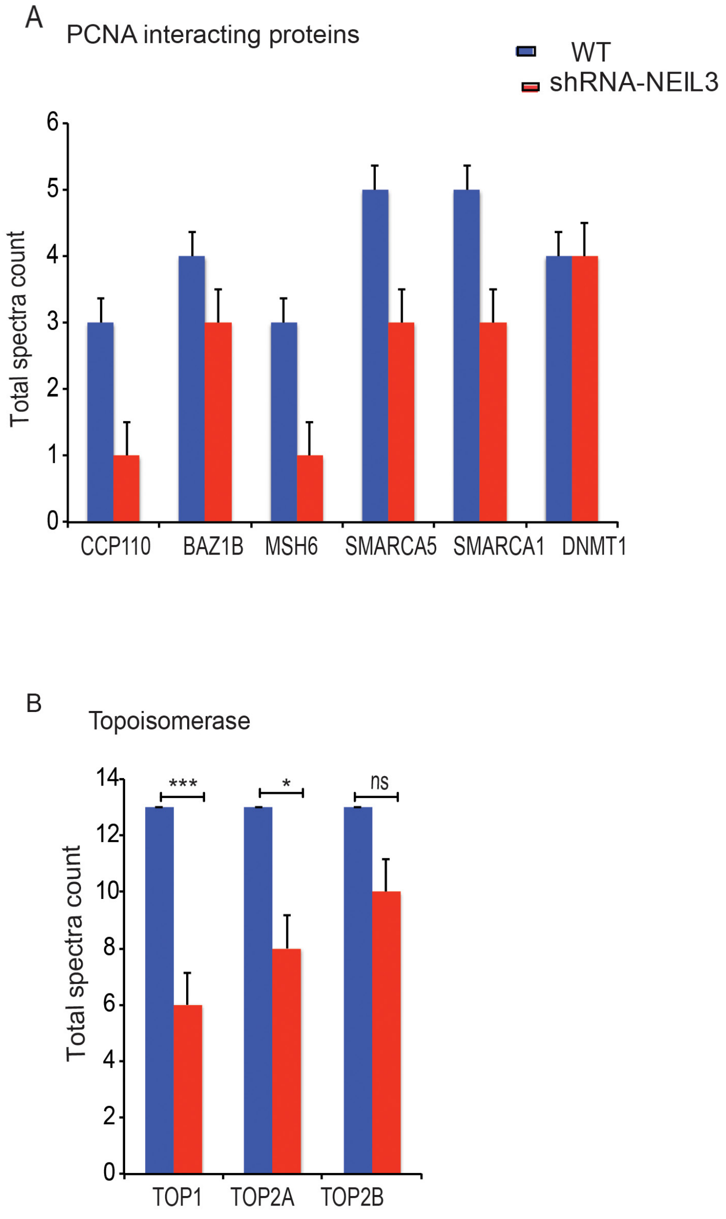

Supplementary Figure 1: NEIL3 is required for the recruitment of proteins to maintain chromatin and topological structure. (A) PCNA-binding partners in nascent DNA after replication fork stall/ collapse; **(B)** Recruitment of topoisomerase enzyme (Top1A, Top2A, and Top2B) at the replication fork in NEIL3-proficient and NEIL3-deficient cells. All data analyzed using GraphPad Prism software. Two-way ANOVA analysis were applied to compare the presence of protein complex at the replication fork between NEIL3 proficient and deficient cells.

Supplementary Table 1: List of proteins that are associated with DNA damage at replication fork. The proteins were identified using LS/MS/MS methods. The spectrum count was applied to identify the relative change in peptide that detected in NEIL3 proficient versus deficient cells. Ninety-nine percent of protein identity used to collect the peptide data. Proteins were identified and searching using Scaffold viewer software.

See Supplementary File 1
