# Supplementary material for: Loss of NEIL3 DNA glycosylase markedly increases replication associated double strand breaks and enhances sensitivity to ATR inhibitor in glioblastoma cells
Source: Oncotarget. 2017 Dec 4;8(68):112942–58. doi: 10.18632/oncotarget.22896 (PMC5762564; doi:10.18632/oncotarget.22896)
Supplement: Supplementary file 2 [file oncotarget-08-112942-s002.pdf]

Protein	Sequence	Spectrum count WT	Spectrum count shRNA-NEIL3
XRCC1	(K)DSPFGLSFVR(F)	1	2
APEX1	(K)ICSWNVDGLR(A)	7	7
DNA ligase 3	(K)SLLTIQEVDEFLLR(L)	3	3
PARP1	(R)VADGMVFGALLPCEECSGQLVFK(S)	26	27
FEN1	(K)LIADVAPSAIR(E)	29	29
NEIL1	RTRRAKRDLPKRTATGRP	1	1
NEIL3	GPRTLNPDSPRCSKHNRL	2	0
PCNA	(R)NLAMGVNLTSMSK(I)	2	4
Rad51	GFHHTVEAVAYAPKKEL	2	1
Rad50	(R)DSLIQSLATQLELDGFERGPFSER(Q)	1	2
BRCA1	(K)LPVDFSNIPTYLLK(D)	1	1
BRCA2	(R)KFAGLLK(N)	1	4
RPA34	GHTVTEPIQPLEPELPGEGQPEAR	1	8
DNA-PK	LLLQGEADQSLLTFIDK	20	9
XRCC5	(R)HLMLPDFDLLEDIESK(I)	32	39
XRCC6	AGDLRDTGIFLDLMHLK	28	33
TOP1	(K)EMTNEEKNIITNLSK(C)	13	6
TOP2A	(K)IFDEILVNAADNK(Q)	13	8
TOP2B	(K)AASNcGIVESILNWVK(F)	13	10
CHK1	(R)LLNKMcGTLPYVAPELLK(R)	1	3
ATM	DGLGLIYGVISSTAVSR	1	1
ATR	AEHSGSVNVFMLPSSK	1	3
CDK2	(K)FMDASALTGIPLPLIK(S)	4	3
Mre11	(R)LRVDYSGGFEPFSVLR(F)	3	3
DNA polymerase epsilon subunit 3	(M)AERPEDLNLPNAVITR(I)	1	1
DNA polymerase epsilon subunit A	(R)KLEDYGEQK(S)	2	1
DNA polymerase delta	GDDRVSGFVALVTGTVLK	1	1
MSH2	(R)LFDRGDFYTAHGEDALLAAR(E)	5	6
MSH6	(R)LDAIEDLMVVPDKISEVVELLK(K)	3	1
MCM2	(R)GLALALFGGEPK(N)	7	7
MCM3	(R)ALKDFVASIDATYAK(Q)	10	6
MCM4	(R)FIDPLAKEEENVGIDITEPLYMQR(L)	9	12
MCM5	(R)LAALPNVYEVISK(S	2	2
MCM6	(K)DFYVAFQDLPTR(H)	4	2
MCM7	(K)LFADAVQELLPQYK(E)	12	8
FANCA	(R)LRAVLGHNEDDSSVEISKIQLSINTPR(L)	1	1

FANCE	(K)MESLEPDAQVLMLGQILELPWKEETFLV LQSLLER	1	1
FANCJ	MSSMWSEYTIGGVK(I)	2	1
FANCI	(K)SLELLPIILTALATK(K)	2	4
CCP110	EDSTLQGEVTVLGHTVK	3	1
BAZ1B	(K)LVDTAWLEIMTK(Y)	4	3
SMARCA1	MKDMKGSSNLVDIDK	1	1
SMARCA5	(K)TLQTISLLGYMK(H)	5	3
DNMT1	(K)LPLFPEPLHVFAPR(A)	4	4
